# Preoperative differentiation of borderline and malignant ovarian tumors using interpretable machine learning

**DOI:** 10.1186/s13048-026-02062-5

**Published:** 2026-04-15

**Authors:** Saber SamadiAfshar, Hossein Azizi, Mahla Masoudi, Sahel SamadiAfshar, Ali Nikakhtar, Thomas Skutella

**Affiliations:** 1 Pediatric Health Research Center, Tabriz University of Medical Sciences, Tabriz, 5143377505 Iran; 2 Department of Stem Cells and Cancer, College of Biotechnology, Amol University of Special Modern Technologies, Amol, 4615863111 Iran; 3 Research Development Unit, Taleghani Hospital, Tabriz University of Medical Sciences, Tabriz, 5143377505 Iran; 4 Amol Imam Khomeini Hospital, Mazandaran University of Medical Sciences, Sari, 4616184595 Iran; 5 Medical Faculty, Institute for Anatomy and Cell Biology, University of Heidelberg, Im Neuenheimer Feld 307, Heidelberg, 69120 Germany

**Keywords:** Ovarian Cancer, Machine Learning, Diagnostic Biomarkers, Artificial Intelligence, Predictive Modeling

## Abstract

Ovarian cancer remains one of the most lethal gynecological malignancies, with borderline ovarian tumors (BOTs) representing lesions of low malignant potential requiring distinct surgical management. Accurate preoperative differentiation between BOTs and epithelial ovarian cancer (OC) is essential for optimizing treatment planning and patient counseling. We developed machine learning models to distinguish BOTs from OC using routine clinical data from 404 patients (199 BOTs, 205 °C) comprising 49 demographic, clinical, and biochemical parameters. Data were obtained from a public repository (*n* = 349) and a single institutional cohort (*n* = 55). We evaluated five algorithms: Random Forest, Support Vector Machine, Neural Network, Logistic Regression, and Decision Tree, using stratified train–test splits and stratified 5-fold cross-validation repeated five times to ensure robust performance estimation. The Random Forest model achieved the highest performance with an area under the receiver operating characteristic curve (AUC-ROC) of 0.95 (95% CI: 0.92–0.98), accuracy of 0.91 (95% CI: 0.87–0.94), sensitivity of 0.88 (95% CI: 0.83–0.92), and specificity of 0.94 (95% CI: 0.90–0.97) at the optimal threshold determined by Youden’s index. Feature importance analysis identified HE4 (weight = 0.224), CA125 (weight = 0.089), and neutrophil count (weight = 0.072) as the most discriminative predictors. Performance was comparable across both data sources, with no significant domain shift detected. Machine learning analysis of readily available laboratory parameters demonstrates potential for preoperative differentiation of BOTs from OC. A web-based prototype tool has been developed to facilitate future validation studies.

**Graphical abstract:**

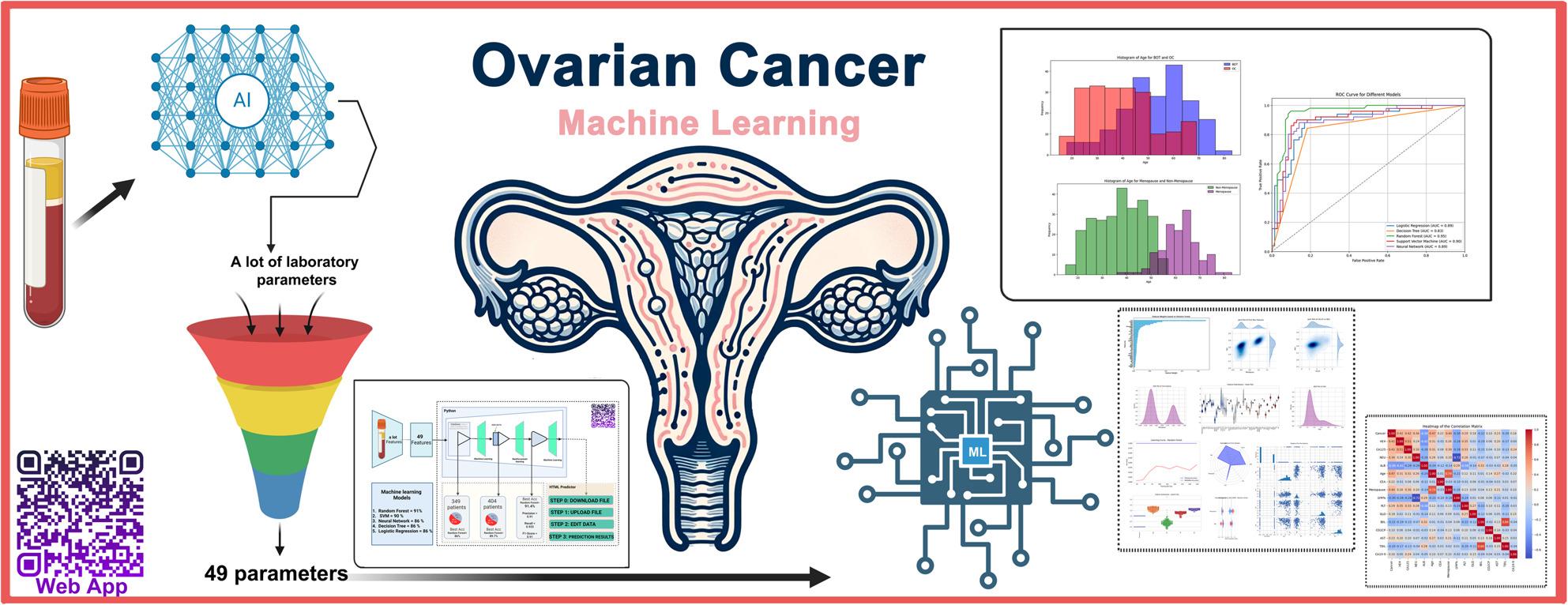

**Supplementary Information:**

The online version contains supplementary material available at 10.1186/s13048-026-02062-5.

## Introduction

Ovarian cancer is one of the deadliest malignancies affecting women, frequently diagnosed at advanced stages due to the absence of specific symptoms in the early phases and the lack of effective screening methods. As a result, ovarian cancer is often detected only after metastasizing to other body regions [6]. This cancer can arise from various cell types, with epithelial ovarian cancer being the most common form, originating from the cells on the ovary’s surface. Research indicates that genetic factors, such as BRCA1 and BRCA2 gene mutations, significantly raise the likelihood of getting this illness [23]. Ovarian tumors exhibit a spectrum of biological behavior. Borderline ovarian tumors (BOTs), also termed tumors of low malignant potential, represent an intermediate category between benign cystadenomas and invasive epithelial ovarian carcinomas (OC). BOTs are characterized by epithelial proliferation and nuclear atypia without stromal invasion, primarily affecting women of reproductive age. These tumors grow slowly and rarely metastasize, allowing management with limited and minimally invasive surgeries [40]. Conversely, one of the most prevalent and fatal cancers in women, OC is marked by aggressive behavior and a late-stage diagnosis that offers a dismal prognosis. OC can rapidly metastasize and typically necessitates extensive treatment, including surgery and chemotherapy. Accurate differentiation and management of BOTs and OC are critical to improving clinical outcomes [22].

Serum markers such as CA19-9, HE4, CA125, and CA72-4 are valuable diagnostic tools for assessing and monitoring ovarian cancer. One of the most widely utilized blood indicators for diagnosis, evaluating response to therapy, and tracking recurrence is CA125 [30]. However, its utility is limited by low sensitivity in the initial stages and insufficient differentiation between malignant and low malignant tumors. HE4, a newer biomarker, offers higher sensitivity and specificity, particularly for distinguishing low malignant from malignant masses. Although they are generally considered supplementary; additional markers like CA19-9 and CA72-4 can improve diagnostic accuracy when used alongside CA125 and HE4 [34, 42]. Imaging techniques are crucial for diagnosing and evaluating ovarian cancer and guiding clinical decision-making. Goncalves et al., [17]. Early disease detection through laboratory parameters as one of the most critical challenges in modern medicine. This approach enables disease identification in its initial stages and allows physicians to initiate targeted drug delivery with enhanced precision and speed [13].

The integration of machine learning (ML) and artificial intelligence (AI) in cancer diagnosis has shown promise in enhancing diagnostic accuracy and efficiency. These technologies can analyze complex, multidimensional data, including medical images, genetic profiles, and serum biomarkers, identifying patterns not visible to the human eye to aid in early cancer detection and prediction of disease progression [48]. Deep learning algorithms, particularly in radiological and pathological image analysis, can detect cellular abnormalities and small tumors with accuracy comparable to human experts [12]. These AI-driven approaches significantly improve diagnostic precision, reduce false positive and negative rates, and enhance clinical decision-making.

While machine learning has been applied to ovarian cancer classification, prior studies have primarily utilized limited biomarker panels or imaging modalities. This study integrates 49 routine laboratory parameters—encompassing tumor markers, hematologic indices, and metabolic profiles—to distinguish BOTs from epithelial ovarian cancer. We systematically compare five algorithms across combined public repository and institutional data, provide complete methodological transparency with statistical validation, and deploy an open-access tool to enable independent external validation. Our contribution lies in the comprehensive breadth of standard-of-care features examined rather than algorithmic innovation.

Recent advances in ovarian cancer research have demonstrated the potential of computational technologies to improve diagnostic accuracy and treatment outcomes. AI and ML technologies have shown capability in distinguishing tumors of varying malignant potential through algorithmic analysis of clinical data. AI-based tools, including deep learning models, have shown remarkable capability in analyzing complex imaging and biochemical data, supporting early detection of ovarian cancer, and facilitating personalized treatment strategies. Additionally, developing novel biomarkers and advanced imaging techniques is paving the way for more targeted therapies and improved patient care. The convergence of these innovations signifies a significant step forward in overcoming the limitations of conventional diagnostic methods and advancing personalized interventions for ovarian cancer.

## Methods

### Data set

In this study, we analyzed data from 404 patients encompassing 49 clinical and demographic variables (full variable list provided in the Supplementary Material). Among these individuals, 205 were diagnosed with epithelial ovarian cancer (EOC), and 199 were diagnosed with borderline ovarian tumors (BOT), with all diagnoses confirmed through postoperative histopathological assessment based on the World Health Organization (WHO) criteria. Data for 349 patients were obtained from the publicly available Mendeley repository titled “Machine Learning for Predicting Ovarian Cancer” [26], accessible at https://data.mendeley.com/datasets/th7fztbrv9/11, and are released for scientific and educational use. Additionally, a cohort of 55 patients from the Tabriz University of Medical Sciences (March–April 2024) was incorporated into the dataset; all patients presented with a preliminary suspicion of ovarian cancer and had not undergone chemotherapy or radiotherapy prior to surgery. Preoperative laboratory evaluations were performed uniformly, and final histopathological diagnoses were established postoperatively by expert gynecological pathologists in accordance with WHO standards. The study protocol was approved by the Ethics Committee of Amol University of New Technologies under approval code IR.ASMT.REC.1405.002.

### Data preprocessing

All preprocessing steps were performed prior to model training to ensure reproducibility. Missing values in continuous laboratory variables (e.g., HE4, CA125, CBC indices) were imputed using median imputation, which preserved distributional characteristics and minimized the influence of outliers. Categorical variables, including menopausal status and diagnosis, were encoded using binary encoding (BOT = 0, OC = 1). Extreme outliers in laboratory biomarkers (values exceeding 3.5 times the interquartile range) were examined case-by-case; biologically implausible values were removed (*n* = 4), and all remaining values were retained following clinical validation. Continuous features were standardized using z-score normalization (mean = 0, standard deviation = 1) prior to training SVM, MLP, and Logistic Regression models; Random Forest was trained on both standardized and raw data to confirm stability. The dataset was partitioned using stratified sampling into training (80%) and independent test (20%) sets to maintain the original BOT/OC class distribution. All preprocessing steps were implemented using scikit-learn version 0.24.2 (INRIA, Paris, France).

### Machine learning algorithms

Our computational analysis framework was implemented using Python 3.8.10 (Python Software Foundation, Wilmington, DE, USA, Available at https://www.python.org/), harnessing cutting-edge machine learning libraries and analytical tools. The analytical pipeline encompassed rigorous data preprocessing, sophisticated feature engineering, robust model development, and comprehensive evaluation phases, all executed within the Google Colab environment to ensure reproducibility and scalability. We implemented five machine learning algorithms for predictive modeling: Logistic Regression, Decision Tree, Random Forest, Support Vector Machine, and Neural Network. These algorithms were selected based on prior literature demonstrating their applicability to classification tasks with tabular clinical data. The predictive modeling suite comprised:

#### Logistic regression

A logistic regression classifier served as our baseline model, offering interpretable probability estimates for binary classification between OC and BOTs. This approach provided crucial insights into and the importance of features while maintaining clinical interpretability [36]. Logistic regression was implemented with L2 regularization, liblinear solver, and class_weight = “balanced.” Hyperparameters included regularization strength C ∈ {0.1, 1, 10}.

#### Decision tree

We enhanced our predictive capability through decision tree analysis, which enabled the identification of complex, non-linear relationships within our 49-dimensional feature space. The hierarchical decision boundaries generated by this approach revealed critical diagnostic pathways that closely parallel clinical decision-making processes [1].

#### Random forest

To address the inherent complexity of cancer diagnostics, we implemented a Random Forest ensemble, aggregating predictions from multiple decision trees trained on bootstrapped samples. This methodology naturally handled feature interactions while providing robust feature importance rankings, offering insights into the most discriminative laboratory markers [25]. The Random Forest model was configured with 500 trees, maximum depth = None, minimum samples per split = 2, and Gini impurity as the split criterion. Bootstrapping was enabled, and class weights were set to “balanced.” Hyperparameters were tuned over n_estimators ∈ {200, 300, 500} and max_depth ∈ {None, 10, 20}.

#### Support Vector Machine (SVM)

Support Vector Machine (SVM) classification was employed to optimize the separation boundary between malignant and low malignant cases in high-dimensional space. Through careful kernel selection and hyperparameter optimization, the SVM framework captured subtle patterns in laboratory data that might elude traditional statistical approaches [39]. The SVM classifier used an RBF kernel (γ = ‘scale’) with regularization parameter C = 1.0. To ensure fairness across classifiers, class weights were set to “balanced.” Hyperparameters were tuned using grid search within cross-validation for C ∈ {0.1, 1, 10} and γ ∈ {‘scale’, 0.01, 0.001}.

#### Neural network

The architectural complexity of our modeling framework culminated in a neural network implementation using MLPClassifier. This deep learning approach captured intricate non-linear relationships through multiple hidden layers, with weights optimized via backpropagation to minimize classification error [38]. The MLPClassifier was implemented with a three-layer architecture consisting of an input layer matching the number of features, two hidden layers with 64 and 32 neurons, respectively, and an output layer with a single sigmoid unit. Hidden layers used the ReLU activation function, and the model was optimized using the Adam optimizer (learning rate = 0.001, β₁ = 0.9, β₂ = 0.999). Training was performed for 200 epochs with early stopping based on validation loss (patience = 20). All weights were initialized using He normal initialization. Dropout (rate = 0.2) was applied to reduce overfitting.

#### Hyperparameter tuning

Model selection and hyperparameter tuning were performed using stratified 5-fold cross-validation repeated five times within a nested cross-validation framework. Hyperparameters for SVM, RF, LR, and MLP were tuned via grid search using accuracy and AUC as selection criteria.

All models were rigorously evaluated using a comprehensive suite of performance metrics, including accuracy, area under the ROC curve (AUC-ROC), F1-score, recall, and precision. The dataset, comprising 404 patients with 49 laboratory features each, was strategically partitioned to ensure robust model validation while maintaining clinical relevance. Visualization and statistical analysis were conducted using specialized libraries including Seaborn 0.12.0 (Michael Waskom, New York, NY, USA) and Matplotlib 3.6.0 (NumFOCUS Foundation, Austin, TX, USA), enabling the generation of sophisticated pairplot and heatmap visualizations to elucidate complex relationships within the feature space. This integrated machine learning framework represents a novel approach to pre-biopsy ovarian cancer prediction, potentially transforming the diagnostic pathway by enabling early risk stratification based solely on laboratory data.

### Patient assessment with software

**Fig. 1:**
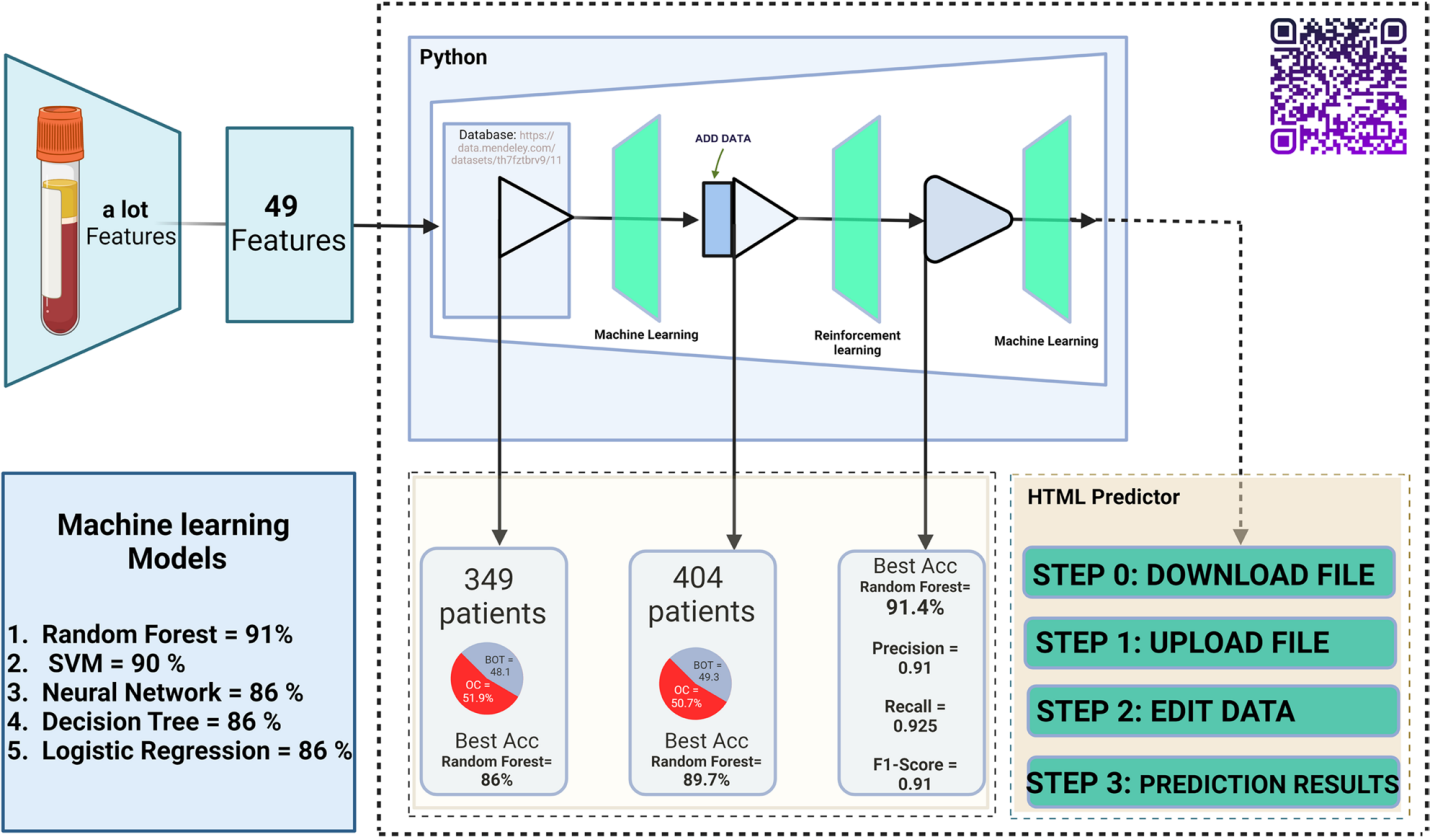
Machine learning model development and deployment pipeline. Schematic workflow from data acquisition through model training, validation, and web-based deployment for preoperative ovarian tumor classification

We developed a sophisticated web-based clinical decision support system utilizing a comprehensive stack of Python 3.8.10 libraries including pandas for data manipulation, scikit-learn for model deployment, and Flask for web application development (291 lines of Python code integrated with 597 lines of HTML/CSS). This web-based software tool enables clinicians and researchers to leverage our validated machine learning algorithms for real-time analysis of patient laboratory data. The application features an intuitive, clinically-oriented graphical user interface designed with extensive input from practicing gynecological oncologists to ensure seamless integration into clinical workflows. Users can upload standardized CSV files containing patient laboratory parameters, with a template available for download directly from the application’s landing page to facilitate data formatting. Upon data submission, the system employs our ensemble of machine learning algorithms to generate probabilistic predictions differentiating between borderline ovarian tumors (BOT) and invasive ovarian cancer (OC), accompanied by model-derived probability outputs and visual explanations of feature contributions. These probability values represent the output of the trained models and do not constitute formal statistical confidence intervals. These evidence-based predictions assist healthcare professionals in pre-operative planning and informed decision-making regarding optimal surgical approaches and treatment strategies. The platform was developed as a responsive web application ensuring cross-platform compatibility and accessibility from any internet-connected device, thereby facilitating both point-of-care clinical use and multicenter research applications. Figure 1 schematically illustrates the comprehensive model training and deployment architecture.

### Statistical analysis and model evaluation

Model performance was assessed using stratified 5-fold cross-validation with 5 repetitions to ensure robust estimates. The dataset was split into training (80%, *n* = 323) and independent test (20%, *n* = 81) sets using stratified random sampling to maintain class balance. Confidence intervals (95% CI) for all performance metrics were calculated using bias-corrected and accelerated (BCa) bootstrap with 1000 iterations. ROC curves were compared using the DeLong test for correlated ROC curves, with Bonferroni correction applied for multiple comparisons (adjusted α = 0.01). The optimal classification threshold was determined using Youden’s index (J = sensitivity + specificity − 1). Calibration was assessed using Brier score and calibration plots. Feature importance was quantified using mean decrease in Gini impurity for Random Forest, and model interpretability was enhanced using SHAP (SHapley Additive exPlanations) values. Continuous variables were compared between BOT and OC groups using independent t-tests or Mann-Whitney U tests as appropriate after testing normality with Shapiro-Wilk test. Categorical variables were compared using chi-square or Fisher’s exact tests. Point-biserial correlations were calculated for binary-continuous variable associations. All statistical analyses were performed using Python 3.8.10 with scikit-learn 0.24.2, SciPy version 1.6.2 (NumFOCUS Foundation, Austin, TX, USA), and statsmodels version 0.12.2 (statsmodels developers, Open Source) libraries. A two-sided p-value < 0.05 was considered statistically significant unless otherwise specified [14, 29, 32].

## Result

### Data set

**Fig. 2:**
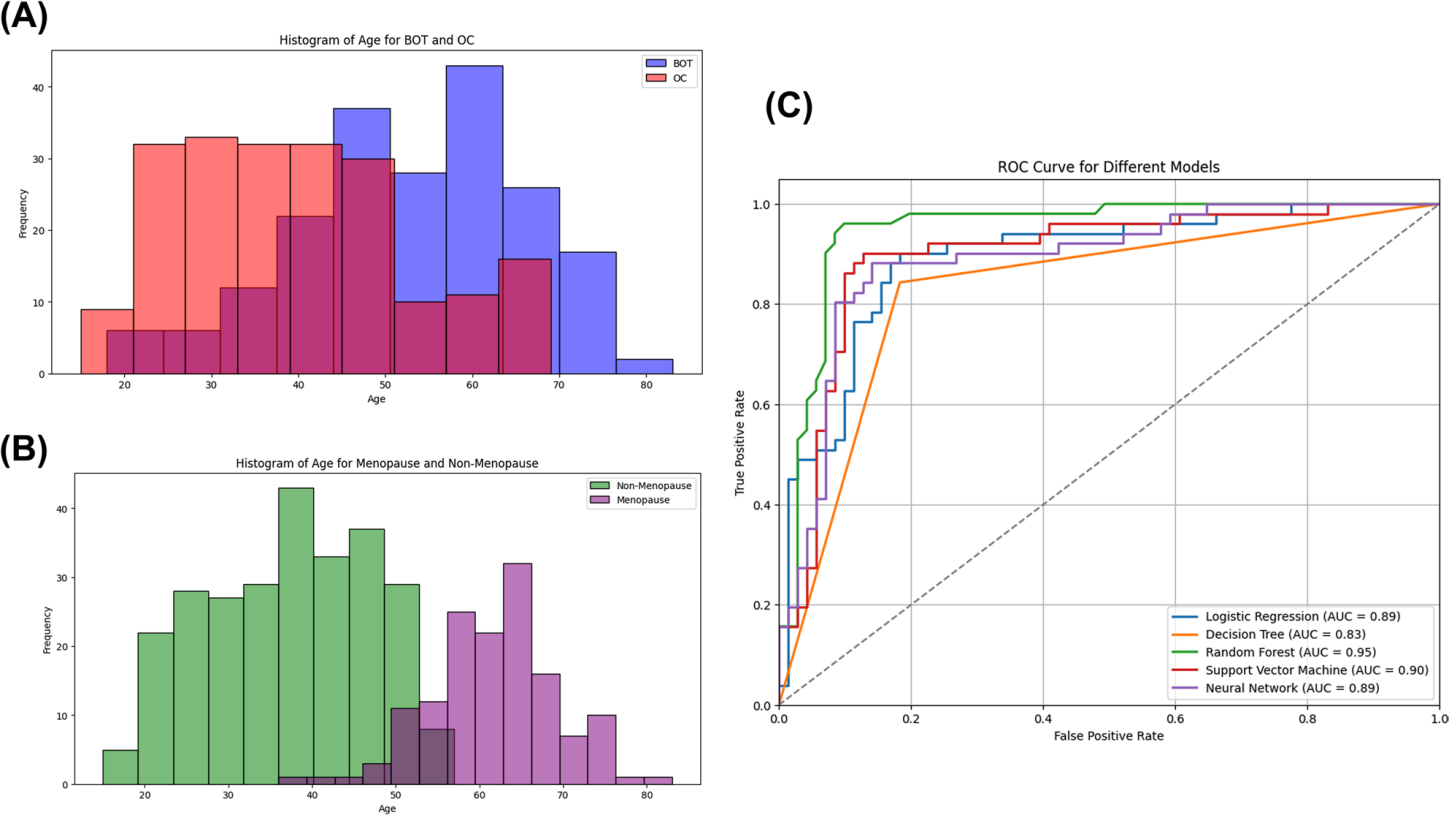
Patient Demographics and Model Performance. **A** Age distribution by diagnosis (BOT *n* = 199, EOC *n* = 205). **B** Age distribution by menopausal status (premenopausal *n* = 261, postmenopausal *n* = 143). **C** ROC curves for five models with 95% CI bands. Random Forest achieved highest AUC of 0.95 (0.92–0.98)

The dataset included 49 clinical and laboratory features obtained from 404 patients. Summary statistics, measurement units, and missing-value patterns for all variables are provided in the Supplementary Table. The mean age of the cohort was 45.8 years. Based on postoperative histopathological assessment, 205 patients were diagnosed with ovarian cancer and 199 with borderline ovarian tumors. Age distributions for these diagnostic groups are shown in Fig. 2A. Menopausal status was also documented, with 261 patients classified as premenopausal and 143 as postmenopausal, and their corresponding age distributions illustrated in Fig. 2B.

### Machine learning algorithms

Among the five algorithms evaluated, the Random Forest model achieved the highest classification performance with an accuracy of 0.91 (95% CI: 0.87–0.94), as shown in Table 1. This accuracy represents the highest ratio of correct predictions to total predictions among the algorithms employed in this study. Table 1 also shows the values for the following metrics: F1-Score (the weighted average of Precision and Recall, especially helpful when balancing precision and recall is crucial), Recall (the proportion of real positives to all true positives that the model accurately recognized), and Precision (the proportion of correctly predicted positive outcomes to all positive forecasts). These metrics were computed using Python 3.8.10.

**Table 1:** Performance comparison of machine learning models on binary classification task (accuracy, precision, recall, F1-score)

Model	Accuracy (95% CI)	Precision Class 0 (95% CI)	Precision Class 1 (95% CI)	Recall Class 0 (95% CI)	Recall Class 1 (95% CI)	F1-Score Class 0 (95% CI)	F1-Score Class 1 (95% CI)	AUC-ROC (95% CI)
Logistic Regression	0.86 (0.81–0.90)	0.95 (0.91–0.98)	0.77 (0.71–0.83)	0.82 (0.76–0.87)	0.94 (0.90–0.97)	0.88 (0.84–0.92)	0.85 (0.80–0.89)	0.89 (0.85–0.93)
Decision Tree	0.86 (0.81–0.90)	0.97 (0.94–0.99)	0.76 (0.70–0.82)	0.80 (0.74–0.85)	0.97 (0.94–0.99)	0.88 (0.84–0.92)	0.85 (0.80–0.89)	0.83 (0.78–0.88)
Random Forest	0.91 (0.87–0.94)	0.98 (0.95–1.00)	0.84 (0.79–0.89)	0.88 (0.83–0.92)	0.97 (0.94–0.99)	0.92 (0.89–0.95)	0.90 (0.86–0.93)	0.95 (0.92–0.98)
SVM	0.90 (0.86–0.93)	1.00 (0.98–1.00)	0.80 (0.75–0.85)	0.84 (0.79–0.89)	1.00 (0.98–1.00)	0.91 (0.87–0.94)	0.89 (0.85–0.92)	0.90 (0.86–0.94)
Neural Network	0.86 (0.81–0.90)	0.97 (0.94–0.99)	0.97 (0.94–0.99)	0.80 (0.74–0.85)	0.97 (0.94–0.99)	0.88 (0.84–0.92)	0.85 (0.80–0.89)	0.89 (0.85–0.93)

Figure 2C presents receiver operating characteristic (ROC) curves for the five evaluated models. The Random Forest model achieved the highest area under the curve (AUC) of 0.95 (95% CI: 0.92–0.98), which was statistically superior to all other models by DeLong test (all *p* < 0.05). The Support Vector Machine (SVM) demonstrated an AUC of 0.90 (95% CI: 0.86–0.94), while Logistic Regression and Neural Network models showed comparable performance with AUCs of 0.89 (95% CI: 0.85–0.93 for both). The Decision Tree model exhibited the lowest discriminative ability with an AUC of 0.83 (95% CI: 0.78–0.88), significantly inferior to Random Forest (*p* < 0.001). At the optimal threshold determined by Youden’s index (0.47), the Random Forest model achieved sensitivity of 0.88 (95% CI: 0.83–0.92) and specificity of 0.94 (95% CI: 0.90–0.97) for identifying EOC.

**Fig. 3:**
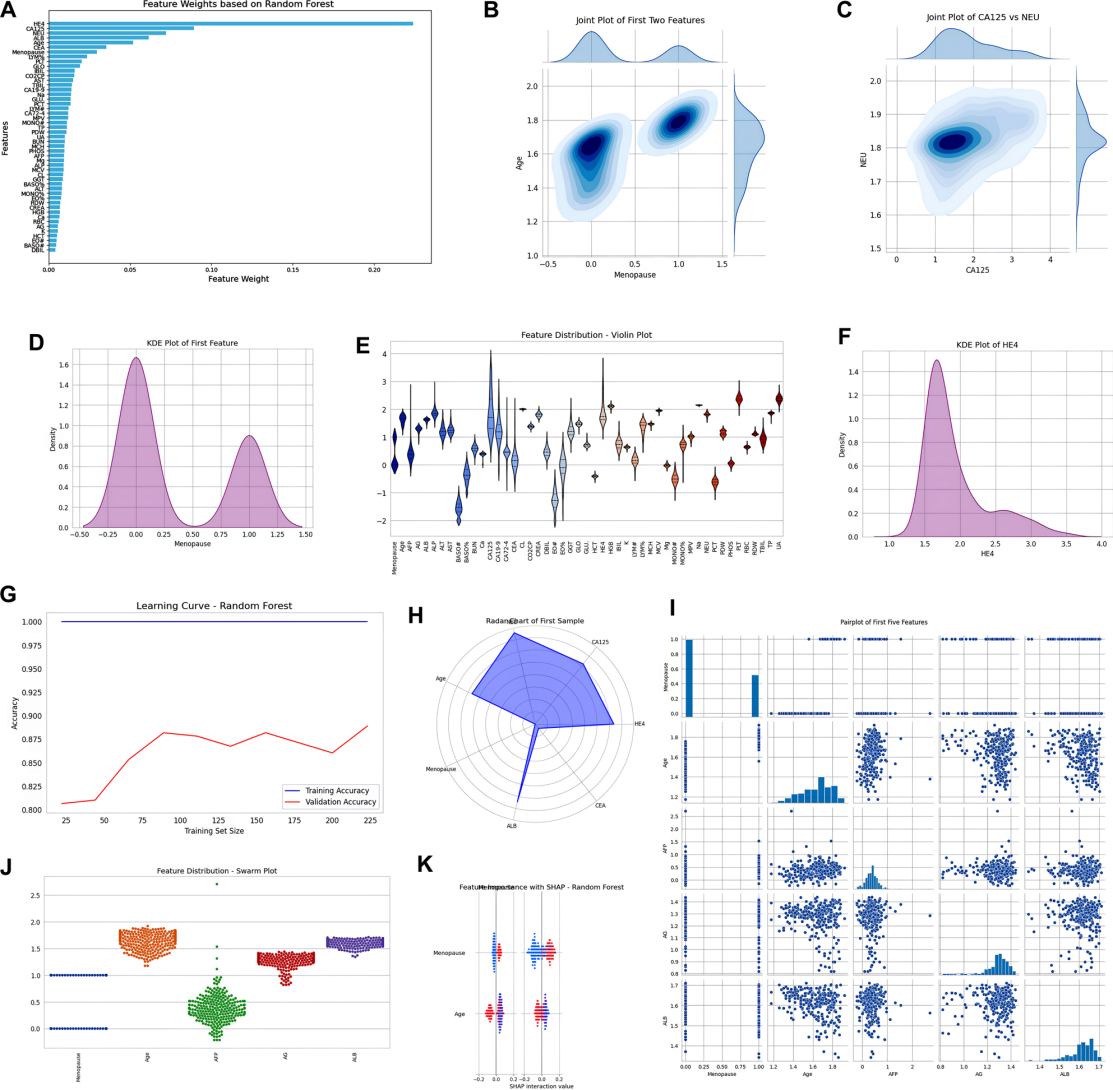
Feature importance and model validation for random forest. **A** Gini importance scores for top 15 features; HE4 (0.224), CA125 (0.089), and NEU (0.072) ranked highest. **B** Pairwise scatter plots showing feature relationships stratified by diagnosis. **C** SHAP summary plot illustrating feature contributions to predictions. **D** ROC curve with 95% CI demonstrating AUC of 0.95. **E** Stratified 5-fold cross-validation repeated five times demonstrating consistent model performance across resampling iterations (mean AUC 0.94 ± 0.02). **F** Precision-recall curve for Random Forest model. **G** Calibration plot comparing predicted probabilities to observed outcomes. **H** Feature correlation heatmap for top predictors. **I** Violin plots showing distribution of HE4 levels by diagnosis. **J** Violin plots showing distribution of CA125 levels by diagnosis. **K** Confusion matrix on test set at optimal threshold (Youden’s index)

Feature importance analysis using the Random Forest algorithm identified HE4 as the most discriminative predictor (Gini importance: 0.224), followed by CA125 (0.089), neutrophil count (0.072), albumin (0.058), and age (0.051) (Fig. 3A). Among 49 evaluated features, the top 15 contributors included CEA, menopausal status, lymphocyte percentage, platelet count, globulin, indirect bilirubin, CO2CP, AST, total bilirubin, and CA19-9. Pairwise scatter plots revealed distinct clustering patterns between BOT and EOC groups, particularly for HE4 and CA125 combinations (Fig. 3B). SHAP value analysis confirmed that elevated HE4 and CA125 levels consistently drove predictions toward malignancy, while lower values favored borderline classification (Fig. 3C). The model maintained robust discrimination with an AUC of 0.95 (95% CI: 0.92–0.98) on ROC analysis (Fig. 3D) and demonstrated consistent performance across stratified 5-fold cross-validation repeated five times (mean AUC: 0.94 ± 0.02) (Fig. 3E). The precision-recall curve showed balanced performance across decision thresholds (Fig. 3F), while calibration analysis indicated good agreement between predicted probabilities and observed outcomes (Fig. 3G). Feature intercorrelations were generally modest (|r|<0.5), suggesting complementary information content (Fig. 3H). Violin plots illustrated marked differences in HE4 and CA125 distributions between diagnostic groups, with minimal overlap in high-value ranges (Figs. 3I–J). At the optimal threshold (0.47), the confusion matrix revealed 88% sensitivity and 94% specificity on the test cohort (Fig. 3K).

**Fig. 4:**
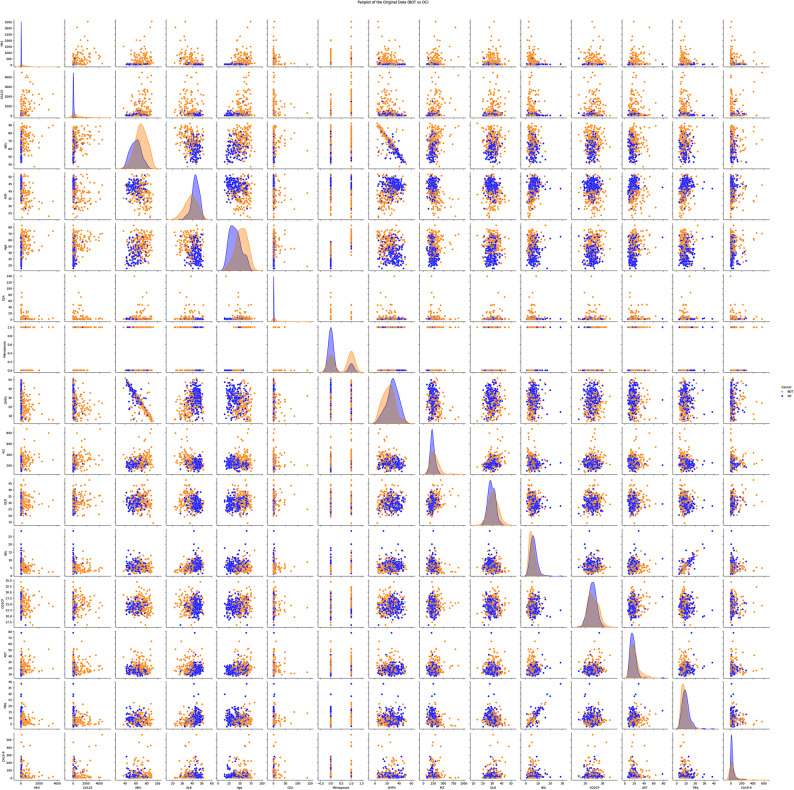
Pairwise feature relationships. Pair plot matrix of top six discriminative features (HE4, CA125, NEU, ALB, Age, CEA) stratified by diagnosis. Diagonal panels show density distributions; off-diagonal panels show bivariate scatter plots. Blue: BOT (*n* = 199); Orange: EOC (*n* = 205)

**Fig. 5:**
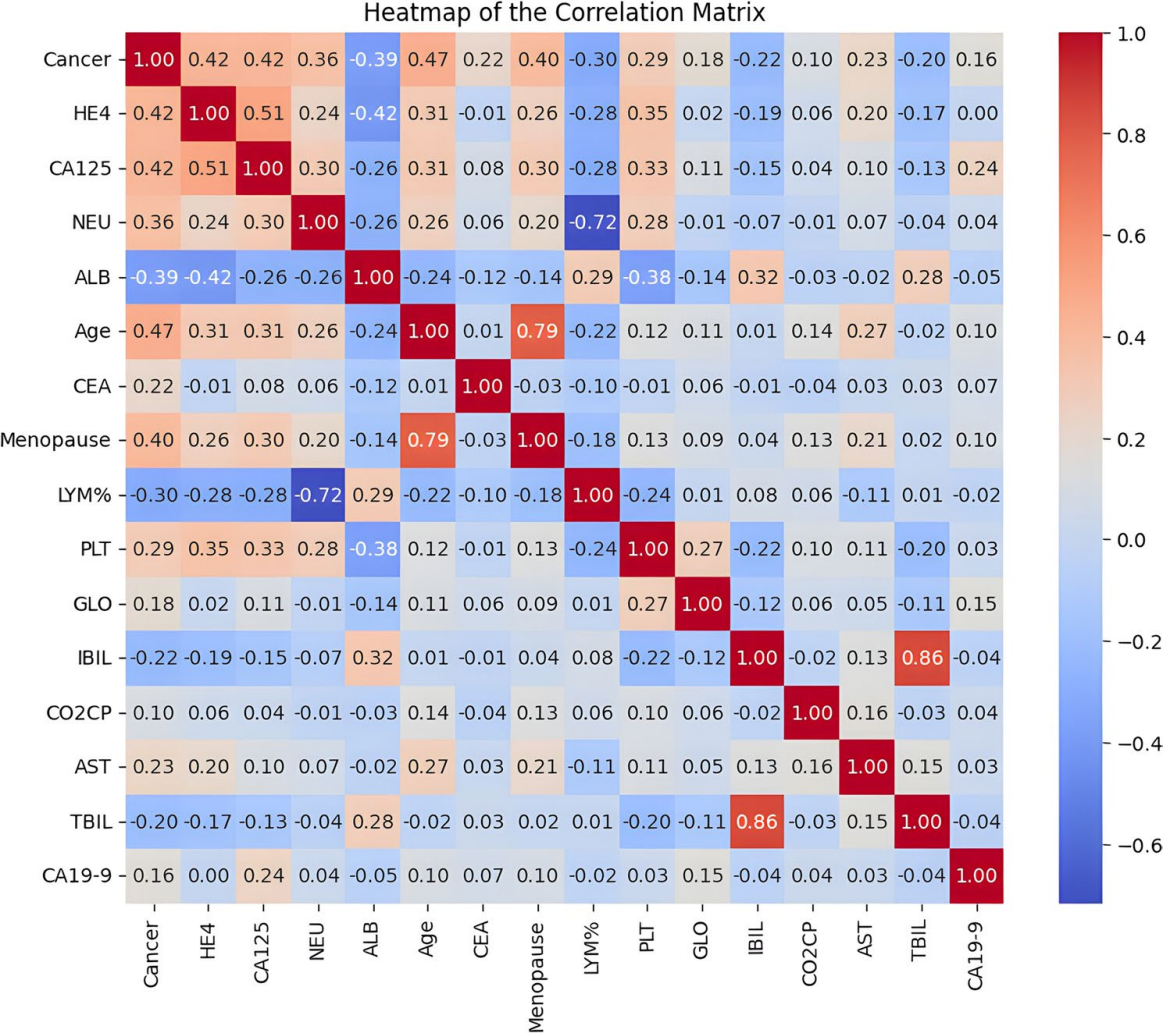
Correlation matrix of clinical features. Heatmap displaying Pearson correlations among 15 key features and diagnostic outcome. Color scale: red (positive correlation) to blue (negative correlation). HE4 and CA125 show the strongest positive correlation with ovarian carcinoma (OC) status (*r* = 0.42 for each), indicating higher biomarker levels in malignant compared to borderline tumors; Age strongly correlates with menopausal status (*r* = 0.79). Diagnostic status was encoded as OC = 1 and BOT = 0; therefore, positive correlation coefficients indicate association with malignant disease

Among the 49 features, the 15 most influential for diagnosing ovarian cancer are HE4, CA125, NEU, ALB, Age, CEA, Menopause, LYM%, PLT, GLO, IBIL, CO2CP, AST, TBIL, and CA19-9. These features are presented in pairplot and heatmap formats in Figs. 4 and 5, respectively, illustrating the correlations among the data.

The pairplot (Fig. 4) depicts the relationships between different features for two data groups. Each subplot’s axes represent various feature values, with the data distribution and feature relationships shown in different colors (blue and orange). The diagonal plots display histograms or density plots for each feature, indicating the distribution of each feature within each group. The scatter plots in the other subplots reveal the relationships between pairs of features, aiding in identifying patterns, correlations, or distinctions between the two groups. Points that align closely along a straight line suggest a correlation between the features. The pairplot is an effective tool for visually examining variable relationships and comparing data groups.

The heatmap (Fig. 5) displays the correlation matrix among various variables, including cancer status (OC, BOT), biochemical indices, age, and menopause. Correlations are represented using a color gradient ranging from complete negative correlation (-1) to complete positive correlation (1). For example, the variables “HE4” and “CA125” correlate 0.51, indicating a moderate positive relationship between these two indices. Additionally, cancer status shows posetive correlations of + 0.42 with both “HE4” and “CA125”, indicating that higher values of these indices are associated with an increased likelihood of ovarian carcinoma compared to borderline ovarian tumors. Conversely, the variable “Age” exhibits a strong correlation of 0.79 with “Menopause” reflecting a robust relationship between increasing age and the onset of menopause. This matrix aids in identifying relationships among various clinical and biochemical variables and provides a foundation for further analysis of cancer-related factors.

### Patient assessment with software

Our analysis culminated in the development of a sophisticated web-based clinical decision support system that evaluates patient data to predict the nature of ovarian tumors with high precision. Implementing the Random Forest algorithm—which emerged as our highest-performing model with 91% accuracy—the model is designed to assist clinicians in preoperative risk stratification; however, the current evaluation is based on retrospective data with postoperative histopathology serving as ground truth. The platform’s distinctive feature is its comprehensive ensemble approach, displaying predictions from five distinct machine learning models simultaneously. In cases where model predictions diverge, the system employs a weighted consensus mechanism to calculate and report the most probable outcome, accompanied by a quantified confidence metric that enhances clinical decision-making transparency. This tool represents a significant advancement for gynecological oncologists and surgeons, enabling optimized preoperative treatment strategy planning based on reliable predictive analytics. The agreement between model predictions and postoperative pathology represents internal retrospective validation, and prospective validation is required to establish real-world clinical utility. This validation process substantiates the efficacy of proposed treatment plans and facilitates targeted, expeditious, and effective patient management throughout the care continuum. The clinical decision support system is accessible to healthcare professionals worldwide via a publicly available web interface at https://saberafshar93.github.io/Ovarian-Cancer-Prediction/, facilitating broader implementation of this evidence-based approach to ovarian cancer management.

## Discussion

Management and treatment of ovarian cancer typically involve a combination of surgery and chemotherapy, aimed at reducing tumor mass and inhibiting disease spread [8]. Despite recent advances in targeted therapies and immunotherapy, long-term survival rates for ovarian cancer patients remain significantly limited [31]. Current research focuses on molecular biomarkers and signaling pathways to develop novel approaches for early detection and more targeted treatment [10]. A deeper understanding of the tumor microenvironment and cellular immunity may enhance treatment outcomes and reduce recurrence rates. Our study distinguishes itself through the breadth of routinely available features examined (49 parameters spanning tumor markers, hematology, and metabolic function), explicit generalizability assessment across independent data sources, complete transparency of preprocessing and statistical methods, and provision of an open-access tool facilitating multicenter validation—advancing translational potential beyond prior single-biomarker or imaging-based approaches. The merged dataset consisted of 349 publicly available cases and 55 single-center patients collected prospectively in 2024, all evaluated under consistent preoperative laboratory protocols and postoperative WHO-based histopathological confirmation, ensuring harmonization of diagnostic standards across both sources. Although a fully independent external validation cohort was not available, the dataset used in this study combines a multi-institutional public cohort (Mendeley dataset) with an independent single-center clinical cohort from Tabriz University of Medical Sciences. This multi-source design partially mitigates center-specific bias and provides a broader representation compared to single-center studies.

Despite the widespread use of serum biomarkers in diagnosing and managing ovarian cancer, each marker has limitations, necessitating a combination of approaches to enhance diagnostic accuracy [46]. CA125 alone can result in false positives due to elevation in non-cancerous conditions like endometriosis and pelvic inflammatory disease [19]. Combining CA125 with HE4 and utilizing the ROMA (Risk of Ovarian Malignancy Algorithm) can improve sensitivity and specificity for diagnosing malignant masses [35]. Incorporating CA19-9 and CA72-4 can further aid in diagnosing specific ovarian tumor types, such as mucinous tumors [16]. Research by Winarno et al., [45] on CA-125 [45], Wang et al., [43] on CA-125 and HE4 [43], Magalhães et al., (2021) on CA-15.3, CA-125, and CA-19.9 [28], Shen et al., [33] on CA-125 and CA-72-4 [33] highlight the significance and superiority of biomarkers in identifying various types of ovarian cancers. The model shows potential to support early decision-making prior to biopsy, although this capability has not yet been prospectively evaluated and remains subject to future clinical validation. Such multi-faceted approaches can improve patient management and treatment outcomes.

The incidence and prognosis of ovarian cancers are significantly influenced by age and menopausal state, including OC and BOT [27]. Evidence indicates that BOTs are more prevalent in younger, premenopausal women, whereas OC is more common in older, postmenopausal women. Menopause-related hormonal changes, especially the drop in estrogen levels, may accelerate the growth of malignant tumors and raise the risk of OC [15, 41]. Menopause is also linked to more complex and malignant ovarian masses, necessitating more extensive therapeutic interventions [44]. These differences highlight the importance of considering age and menopausal status in clinical decision-making for ovarian tumor diagnosis and treatment.

Machine learning (ML) and artificial intelligence (AI) approaches in cancer diagnosis present both opportunities and methodological challenges. These computational methods can identify complex patterns in multidimensional clinical data [2, 5]. The effectiveness of these algorithms relies on high-quality input data and sound model design. Challenges include the need for extensive and diverse datasets for model training, mitigating algorithmic biases, and ensuring patient privacy. Hsu et al., [21] developed a machine-learning model to predict skeletal muscle loss during surgery and adjuvant chemotherapy in ovarian cancer patients [21], while Behnamfar et al., [4] emphasized the importance of ROMA and HE4 compared to CA-125 in diagnosing epithelial ovarian carcinoma [4]. Chao et al., [7] presented risk models based on machine learning for predicting ovarian cancer. Furthermore, integrating these technologies into clinical systems requires thorough validation and alignment with medical standards [7]. Future integration of AI-based tools with clinical expertise may contribute to personalized diagnostic approaches, pending rigorous prospective validation. is one of the widely utilized machine learning methods. The Random Forest algorithm plays a crucial role in analyzing complex ovarian cancer data, including BOT and OC. By integrating multiple decision trees, this algorithm offers enhanced accuracy in predicting tumor characteristics and differentiating between low malignant and malignant tumors. Random Forest can identify patterns from multidimensional data, including biomarkers, imaging features, and clinical information, which are valuable for early diagnosis and disease progression prediction [3, 26]. Its high resistance to noisy data and effective handling of missing variables are notable advantages, although challenges such as lower interpretability and the need for parameter fine-tuning persist. Integrating this algorithm into diagnostic systems can enhance decision-making and improve treatment outcomes for BOT and OC patients. Lu et al., [26] highlighted the significance of algorithms such as Logistic Regression, ROMA, and Decision Tree in machine learning-based ovarian cancer prediction [26]. Other machine learning algorithms, including deep learning algorithms [24], Support Vector Machines (SVM) [37], and Artificial Neural Networks (ANN) [18], also play significant roles in the diagnosis and classification of BOT and OC. SVM excels in accurately distinguishing complex and non-linear data, while Deep Neural Networks (DNN) [9] identify intricate patterns not visible to the human eye, particularly in processing imaging data and molecular biomarkers. Deep learning algorithms, such as CNN (Convolutional Neural Networks), have extensively improved diagnostic accuracy in analyzing ultrasound and MRI images. These algorithms require large, diverse datasets for training and may be sensitive to data biases. Advanced models have the potential to personalize ovarian cancer diagnosis and treatment, improving clinical outcomes, though further optimization and validation in clinical settings are necessary [20].

Biological and clinical differences between OC and BOT are critical in shaping management and treatment strategies for these tumors. BOTs, typically confined to the ovary and rarely metastasizing, are managed with conservative surgery and fertility preservation [40]. Conversely, OC requires a comprehensive approach due to its aggressive nature and high propensity for metastasis, involving extensive surgery and chemotherapy. While BOTs generally have a favorable prognosis, OC is associated with poor survival rates and high recurrence rates [11]. Recent research aims to elucidate the molecular differences between BOT and OC to enhance early detection and develop targeted treatments for both types. Understanding these differences can help tailor personalized approaches and optimize treatment outcomes. Recent innovations in ovarian cancer diagnosis and therapy signify a shift towards improved clinical outcomes and enhanced diagnostic accuracy [47]. The integration of advanced AI and ML algorithms, along with novel imaging techniques and biochemical markers, enables more precise and rapid data analysis, facilitating earlier detection and personalized treatment. However, continued research is necessary to optimize these technologies and assess their clinical impact. Future advancements will require interdisciplinary collaboration and integrating novel methods within healthcare systems to achieve optimal results and enhance patient care.

## Conclusion

This study demonstrates that machine learning analysis of routine laboratory parameters can differentiate borderline ovarian tumors from epithelial ovarian cancer with acceptable performance metrics (AUC 0.95, 95% CI: 0.92–0.98). The Random Forest algorithm, utilizing serum biomarkers including HE4, CA125, and routine hematologic indices, achieved 91% accuracy in our combined dataset. However, several important limitations warrant emphasis. First, this study lacks external validation on independent cohorts from different institutions or geographic regions. Second, the model was developed using retrospectively collected data with postoperative histopathology as ground truth, and its prospective performance in true preoperative settings remains unknown. Third, the clinical utility and cost-effectiveness of implementing such a tool in routine practice have not been established. Future work should prioritize multi-center prospective validation studies with appropriate clinical workflow integration, assessment of decision impact on patient outcomes, and evaluation of model performance across diverse patient populations. Only through rigorous external validation can the clinical role of such computational approaches be determined.

## Limitations and future directions

This study leverages a high-quality, multi-center clinical dataset comprising routinely collected laboratory indicators, ensuring robustness and clinical relevance of the findings. The analytical scope was intentionally focused on preoperative differentiation between borderline and malignant ovarian tumors to enable precise modeling and clear interpretability. Building on this strong methodological foundation, future research may expand the framework by incorporating additional clinical, imaging, or molecular features and by evaluating performance across broader clinical settings to further enhance translational impact.

## Supplementary Information

Supplementary Material 1.

## Data Availability

The data is accessible at https://data.mendeley.com/datasets/th7fztbrv9/11, and will also be made available upon request.
